# Pregnancy With Cardiac Arrest in the Emergency Department: Case Report With Review of Literature

**DOI:** 10.7759/cureus.14148

**Published:** 2021-03-27

**Authors:** Gunaseelan R, Sasikumar Mahalingam, Aswin K, Kishen Goel, Anandhi Devendiran

**Affiliations:** 1 Emergency Medicine, Jawaharlal Institute of Postgraduate Medical Education and Research, Puducherry, IND; 2 Emergency Medicine, Apollo Hospitals, Kolkata, IND

**Keywords:** perimortem cesarean section, cardiac arrest, pregnancy, acls, neonatal resuscitation

## Abstract

Cardiac arrest in pregnancy is an uncommon encounter, with the incidence being one in every 12,000 hospital admissions for delivery. Here we present, one such patient and our experience in managing the patient. A 23-year-old, third-trimester pregnant female presented with a history of polytrauma following a road traffic accident. On initial assessment, she was in cardiac arrest. We initiated high-quality cardio-pulmonary resuscitation (CPR) as per advanced cardiac life support (ACLS) protocol. We also performed a perimortem cesarean section within four minutes of cardiac arrest. A male baby was delivered who did not have any signs of life. Neonatal resuscitation was initiated. However, both the mother and the child could not be revived. Cardiac arrest in pregnancy is a unique scenario in resuscitation, and all emergency physicians should know the key highlights in managing such patients. We review some existing literature and pose some queries that are yet to be answered.

## Introduction

Polytrauma is one of the significant causes of mortality in developing countries like India. Initial resuscitation within the golden hour is often missing because of inadequate pre-hospital care, lack of trained personnel and improper interhospital transfers. Nevertheless, when a pregnant lady sustains polytrauma, the result is often a disaster. Not many emergency departments are equipped to resuscitate a pregnant polytrauma victim. In the following case report, we describe the management of one such patient at the emergency department (ED) in a tertiary care hospital in South India with a literature review.

## Case presentation

A 23-year-old, 38-week pregnant lady, the pillion rider, sustained a road traffic accident. The patient was initially treated at a secondary care center and was referred to us after four hours. She was a multiparous lady, gravida three para two, with the current pregnancy due in two weeks. The patient was found to be in cardiac arrest at the triage desk. Cardiopulmonary resuscitation (CPR) was initiated as per advanced cardiac life support (ACLS) protocols and was rushed to the resuscitation area. The cardiac monitor showed pulseless electrical activity (PEA). After three cycles of CPR, the return of spontaneous circulation (ROSC) was achieved. However, after ROSC, the primary survey as per ATLS was carried out. The airway was already secured with an endotracheal tube. Breathing was intact with bilateral equal chest rise with no sign of any chest injury, and the saturation was 100%. Circulation was inadequate with a heart rate of 132/min and blood pressure of 80mmHg systolic. Hence O negative whole blood was transfused, and the blood bank was alerted for massive transfusion protocol. Urgent call-over was given to the duty Obstetrics and Pediatrics team, and fetal ultrasound was done, which showed no cardiac activity.

Meanwhile, the patient went into cardiac arrest, and hence CPR was initiated as per ACLS protocol. Simultaneously arrangements for perimortem C-section were made. After four minutes of CPR, the duty obstetricians proceeded with a perimortem C-section. A lower segment cesarean section was performed with a Pfannenstiel incision. A lower segment incision in the uterus and a term male fetus was delivered who was fully cyanosed with no cardiac activity. A separate team of emergency physicians and pediatricians resuscitated the fetus, whose attempts were unfruitful. The opened uterus and abdominal cavity were packed and left. High-quality CPR was being continued during the whole procedure and after the procedure. CPR was performed for about 35 cycles. However, both maternal and fetal resuscitation went in vain. No ROSC was achieved. Hence we declared both the mother and baby to be dead.

## Discussion

Anatomical and physiological changes of pregnancy and its implications in cardiac arrest

As it becomes the abdominal organ, the gravid uterus pushes the diaphragm up by four centimeters resulting in an upward displacement of the heart and lung. Thus the site of chest compressions should be moved cephalad in a pregnant patient [1]. The lung is also displaced up, resulting in a decreased functional residual capacity (FRC), decreasing the non-hypoxic apnoeic time, necessitating a modified approach to pre-oxygenation and apnoeic oxygenation.

The non-pregnant uterus is a pelvic organ. It poses no threat to the cardiovascular system as the blood supply to the non-pregnant uterus is 50 ml/min (one percent of the cardiac output); whereas, during pregnancy, the uterus gradually grows and becomes an abdominal organ. It compresses the aortocaval system, thus reducing the venous return (preload) and increasing afterload. As a result of this, resuscitation in the traditional supine position can lead to hypotension. Secondly, as the uterus grows in size, the uterus' oxygen consumption increases. Because of the fetal requirement and the placental requirement, the uterine blood flow increases gradually to attain a requirement of 750 ml/min (15% of the cardiac output).

Further, the increase in fetal hemoglobin causes a left shift of the fetus's oxygen dissociation curve, resulting in increased oxygen uptake from the mother. Whereas in the mother, the curve is shifted to the right resulting in the increased requirement of the partial pressure of oxygen in the blood to maintain an equivalent saturation as that of non-pregnant women. Thus the oxygen supply to major organs is in jeopardy even more in a cardiac arrest scenario.

Due to the hormonal effects of pregnancy, there is an increased likelihood of upper airway edema and the friability of the larynx. This results in a difficult laryngoscopy and a difficult airway scenario. Hence all pregnant patients are anticipated to have a difficult airway. Adding to this, these women are also at increased risk of aspiration due to the relaxation of progesterone's lower oesophageal sphincter [2].

Trauma in pregnancy and its implication in cardiac arrest

As with any other patients, polytrauma poses a significant threat to CPR in pregnant patients also. In addition to the typical hemorrhage sites and the analogy "blood on the floor and four more," there is one more potential site of hemorrhage known as the uteroplacental hemorrhage. Hence additional blood loss can occur in a pregnant patient which cannot be easily quantified or identified [3].

An apparent increase in the plasma volume (40%) in comparison to the cells and, in some cases, reduction of platelets (15%) results in dilutional thrombocytopenia results in profuse bleeding even though the traditional labeling of pregnancy as the hypercoagulable state in pregnancy. Trauma is the leading cause of maternal mortality accounting for 46% [4].

Recommendations for managing cardiac arrest in pregnant patients

Immediate call for help. Involve the obstetric team and pediatric team if available. Consider securing a definitive airway early as adequate chest rise with bag-mask ventilation may not be obtainable. Consider securing intravenous access, preferably above the level of the diaphragm. Consider keeping a wedge under the abdomen to avoid aortocaval compression. Or if a wedge is not available, consider manual uterine displacement (Manual uterine displacement can be a one-handed technique with the assistant's left hand displacing the uterus to the left from the right side or two-handed when both of the assistant's hands are used for displacing the uterus to the left). Consider changing the site of chest compressions to one to two inches above the traditional site. Consider using a nasogastric tube early (if possible) as the risk of aspiration can be minimized. Consider perimortem cesarean section if there is no ROSC achieved within five minutes of initiation of CPR. Guidelines state that the perimortem cesarean section should be done at the place of arrest, i.e., either in the pre-hospital setting or in the ED. Hence all emergency physicians should be trained in the procedure [5].

Procedure of perimortem C-section

Equipment required includes no. 10 blade, retractor (if available), Foley's catheter, pad and sponges, neonatal resuscitation devices. If nothing is available in the immediate vicinity, a no. 10 blade is all that is sufficient.

Clean the abdomen with an antiseptic solution. Perform a vertical midline incision from xiphoid to pubic symphysis and perform another vertical incision in the uterus's upper segment [6]. Suppose the bladder is full empty the bladder or retract the bladder. Injury to the bladder is not a significant concern while performing a perimortem cesarean section. After delivering the baby and placenta, pack the uterus with pads and carry on with resuscitation. Closing the uterus and abdominal incision can be done after achieving ROSC or after declaring the patient [7].

Our experience with perimortem C-section as against the recommended guidelines

We used a low transverse (Pfannenstiel) incision against the recommended vertical incision and a lower segment incision in the uterus against the recommended midline vertical [8]. We did not find any difficulty using the lower segment cesarean section to deliver the baby and placenta. Hence we suggest that any incision may be used based on the operator's experience and the available facilities, provided there is no time delay during the lives-saving procedure.

Some important questions yet to be answered?

Some of the questions are - shifting the chest compression site to the traditional site after delivering the baby (as logic would suggest). Administration of uterotonics after delivery of the baby. Cesarean section in the ED even if we achieve ROSC within five minutes for improving the chances of survival of the mother and the fetus. The need for the development of post-ROSC/post-C-section guidelines in a case of pregnancy with cardiac arrest.

## Conclusions

Resuscitation of a pregnant patient is not one of the commonly encountered scenarios in the ED. Neither is trauma in near-term pregnancy. However, with the increasing incidence of motor vehicle accidents, these scenarios could change. Hence, all emergency physicians and primary care providers need to know about ACLS recommendations and modifications for pregnant patients. Also, all emergency rooms should be equipped to do an emergency perimortem C-section, and all emergency physicians must be trained to do the procedures. Serial simulation-based training and training in manikins and involving a senior emergency physician who has handled such scenarios and involving other specialties like obstetrics and pediatricians can increase the chances of successful resuscitation.
